# Supplementary figures and images for: The inflammasome pathway is activated by dengue virus non-structural protein 1 and is protective during dengue virus infection
Source: PLoS Pathog. 2024 Apr 25;20(4):e1012167. doi: 10.1371/journal.ppat.1012167 (PMC11075848; doi:10.1371/journal.ppat.1012167)

A

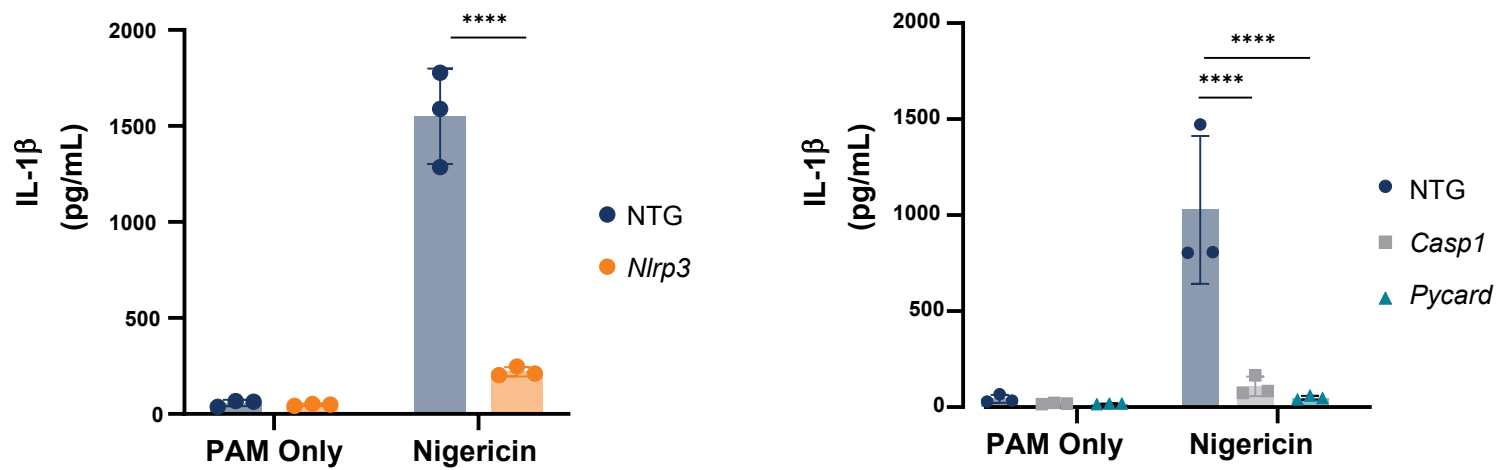

B

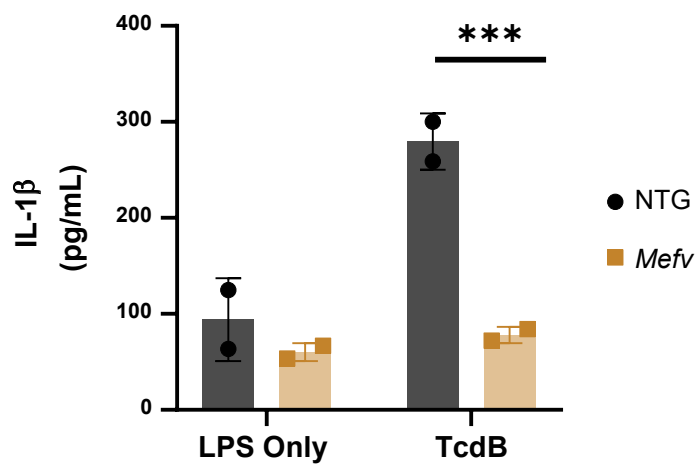

C

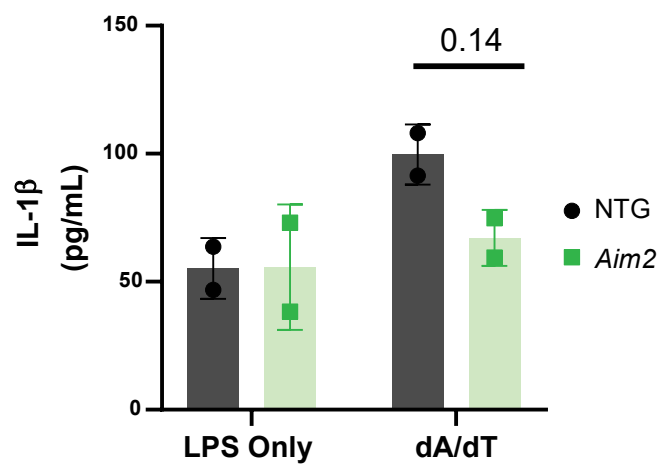

Supplemental Figure 1

Supplement: S1 Fig — (A) BMDMs were nucleofected with Cas9-gRNA ribonuclear protein complexes to knock out the indicated genes. Two gRNAs per gene were used per nucleofection. Knockout BMDMs were primed with PAM3CSK4 (1μg/mL) for 17h and treated with nigericin (5uM) or left untreated for 2h. Supernatants were then collected, and IL-1β levels were measured by ELISA. (B-C) Same as in A, except cells were primed with LPS (5ug/mL) for 4 hours and then treated with TcdB (5ug/mL) (B) or Poly(dA:dT) / LyoVec (5ug/mL) (C) for 24h before collecting supernatant. *p<0.05, **p<0.01, *** p< 0.001, ****p<0.0001, ns (not significant), p> 0.05. Statistical significance was determined using two-way ANOVA with Holm-Sidak’s multiple comparisons test. The data are shown as the mean ± SD of least 2 biological replicates per guide. NTG, non-targeting guide. (PDF) [file ppat.1012167.s001.pdf]

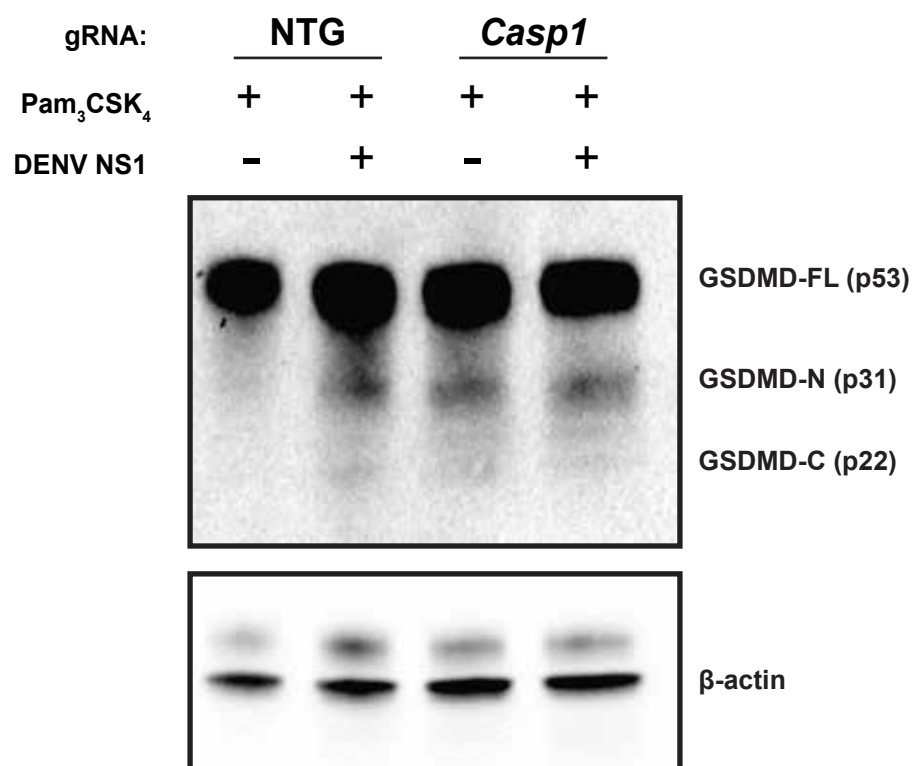

Supplemental Figure 2

Supplement: S2 Fig — Representative Western blots of cell lysates from BMDMs nucleofected with Cas9-gRNA ribonuclear protein complexes targeting Casp1. Nucleofected BMDMs were primed with PAM3CSK4 (1μg/mL) for 17h and then treated for 48h with 10ug/mL DENV2 NS1 or left untreated for 48h without NS1 treatment. A non-targeting guide (NTG) was used as a control. (PDF) [file ppat.1012167.s002.pdf]

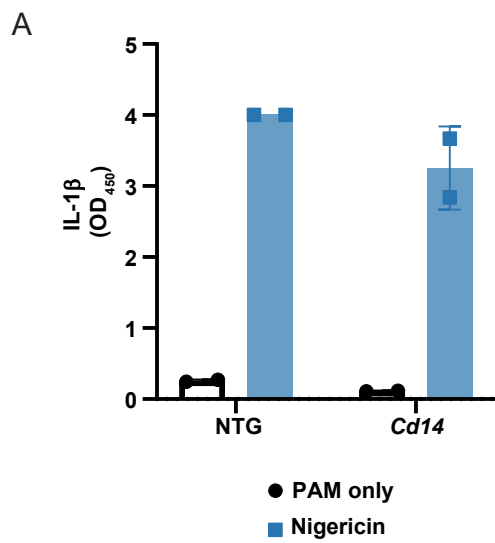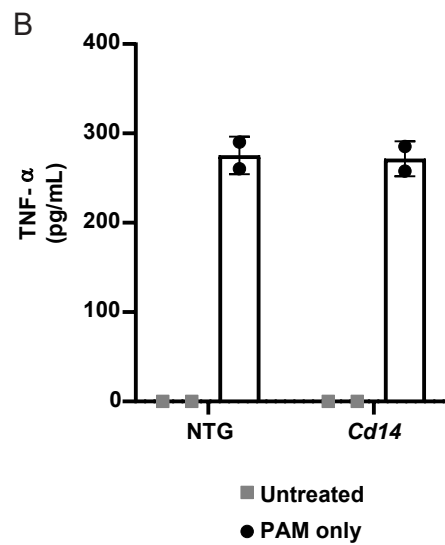

Supplemental Figure 3

Supplement: S3 Fig — (A-B) BMDMs from Fig 3D were primed with PAM3CSK4 (1μg/mL) for 17h and treated with 5μM nigericin or no treatment for 24h. IL-1β levels in supernatant were measured by ELISA (A). TNF-α levels were measured in supernatants 17h post-priming with PAM3CSK4 (B). (PDF) [file ppat.1012167.s003.pdf]
